# Editorial: Innovative approaches for wound treatment

**DOI:** 10.3389/fphar.2025.1738213

**Published:** 2025-12-09

**Authors:** Junxiao Zhang, Shenglin Geng, Guojuan Fan, Jinlong Ma

**Affiliations:** 1 School of Pharmacy, Shandong Second Medical University, Weifang, Shandong, China; 2 Dermatology, Weifang Hospital of Traditional Chinese Medicine, Shandong Second Medical University, Weifang, Shandong, China; 3 Shandong Engineering Research Center for Smart Materials and Regenerative Medicine, Shandong Second Medical University, Weifang, Shandong, China

**Keywords:** wound healing, wound management, foundational research, therapeutic design, antibacterial strategy

Wound healing constitutes a sophisticated biological cascade involving inflammation resolution, tissue regeneration, and extracellular matrix remodeling. Yet it remains a pressing clinical challenge—particularly in refractory cases such as diabetic ulcers, severe burns, and drug-resistant infected wounds (Wang et al., 2023a; Wang et al., 2023b). Against the backdrop of a growing global burden of diabetes, age-related wounds, and traumatic injuries, the development of innovative and translatable wound management strategies has never been more urgent (Wang et al., 2024). This Research Topic brings together 12 selected contributions—comprising 9 original research articles, 2 comprehensive reviews, and 1 thought-provoking opinion piece—each tackling critical unresolved Research Topic in wound care through cutting-edge advances in biomaterial engineering, bioactive compound development, and mechanism-driven therapeutic design.

Two review articles provide foundational frameworks for key research domains in this Research Topic. The first addresses the emerging threat of antibiotic-resistant Cutibacterium acnes by systematically evaluating phytochemicals as promising therapeutic alternatives. It highlights compounds such as resveratrol—a polyphenolic antioxidant derived from plants like Smilax, peanuts, and berries—and punicalagin, detailing antibacterial mechanisms including membrane disruption, lipase inhibition, and redox homeostasis regulation, which collectively offer effective antimicrobial action with reduced adverse effects (Sun et al.). The second review underscores the unique biological properties of silk proteins, sericin and fibroin, which exhibit high biocompatibility, low immunogenicity, and significant immunomodulatory capacities—such as promoting macrophage M2 polarization and modulating cytokine release—thereby supporting their broad applicability in advanced wound dressings and tissue regeneration (Tian et al.).

Complementing these reviews, an opinion piece highlights the broad biomedical potential of cold atmospheric pressure plasma in areas such as sterilization, wound care, and even cancer therapy, attributing its bioactivity to a rich mixture of reactive species, charged particles, and ultraviolet radiation (Li et al.). However, the article also notes a critical limitation in burn wound treatment: the desiccating effect of high gas flow rates and the emission of harmful UV radiation contradict the established principles of moist wound healing, thereby restricting its therapeutic utility.

The Research Topic further presents nine original research articles that introduce innovative, mechanism-driven solutions for wound repair. In the realm of biomaterial engineering, one study reports a novel double-cross-linked PDGA hydrogel (comprising polyacrylamide, dopamine-grafted sodium alginate, glycidyl methacrylate-grafted gelatin, and Angelica sinensis polysaccharide), which rapidly forms *in situ*, acts as a protective barrier, and accelerates healing through rapid hemostasis, enhanced cell proliferation, collagen deposition, and angiogenesis (Liu et al.). Another study combines biomaterials with stem cell-derived exosomes by constructing rIGF1-enriched exosomes delivered via a silk fibroin–collagen hydrogel, effectively promoting annulus fibrosus wound repair and attenuating intervertebral disc degeneration in rats by stimulating cell proliferation and migration (Tian et al.).

To address bacterial infection-related impairments, a multifunctional nanoplatform (P(H)ZPAg) was developed by embedding palladium hydride into a ZIF-8 framework, modifying its surface with polydopamine, and generating silver nanoparticles *in situ*. This system enables synergistic antibacterial action through controlled hydrogen release, photothermal conversion, and Ag^+^ activity, collectively accelerating wound healing in infected models (Wang et al.). Separately, a traditional herbal formula (BaDuShengJi San) was reformulated into a stable carbomer-based hydrogel, which significantly enhanced its antibacterial efficacy (MIC 64 μg/mL), accelerated diabetic wound re-epithelialization by 40%, and reduced its inherent renal toxicity (Zhang et al.).

Beyond biomaterial innovations, several studies focus on bioactive compounds and peptides. Nervonic acid, a key component of myelin and nerve cell membranes, was shown to uniquely synchronize neurogenesis and angiogenesis, enabling holistic repair of both neural and vascular tissues (Liu et al.). Thymoquinone, a natural quinone, enhanced skin flap survival by 44% through dual regulation of SIRT1/NF-κB-mediated pyroptosis and pro-angiogenic activity, providing a synergistic countermeasure to ischemia-reperfusion injury (Yang et al.). The human antimicrobial peptide LL37, the sole cathelicidin-derived peptide in humans, not only exerts broad-spectrum microbicidal and immunomodulatory effects but also acts as a potent angiogenic agent by activating the VEGFA-PI3K/AKT/mTOR pathway, suggesting new translational avenues from antimicrobial to ischemic wound therapy (Yang et al.). Another investigation revealed that narirutin, a citrus-derived flavonoid, accelerates diabetic wound healing by reprogramming macrophage metabolism via the AMPK/Mfn2 axis, shifting energy metabolism from glycolysis to oxidative phosphorylation, promoting M1-to-M2 polarization, and resulting in a 58% improvement in wound closure (Liu et al.).

In preclinical therapeutic development, the combination of sodium houttuyfonate with penicillin G demonstrated strong synergy against MRSA-infected wounds, reducing bacterial load by 91% and lowering key inflammatory cytokines (IL-6, TNF-α) by 50% in rat models, offering a viable alternative for combating drug-resistant infections (Li et al.).

Collectively, this Research Topic offers a holistic perspective on current advances in wound management. The two review articles establish essential background, the opinion piece provides a critical outlook on antibacterial technology, and the nine original research papers deliver actionable, mechanism-based solutions. We extend our gratitude to all contributing authors for their rigorous work and to the editorial team for their dedicated efforts. It is our hope that this Research Topic will foster cross-disciplinary collaboration, accelerate the translation of novel strategies into clinical practice, and, ultimately, improve outcomes for patients suffering from refractory wounds.
